# Oxidative Stress and the Role of NADPH Oxidase in Glaucoma

**DOI:** 10.3390/antiox10020238

**Published:** 2021-02-04

**Authors:** Jennifer C. Fan Gaskin, Manisha H. Shah, Elsa C. Chan

**Affiliations:** 1 Centre for Eye Research Australia, Royal Victorian Eye and Ear Hospital, East Melbourne 3002, Australia; shah.m@unimelb.edu.au (M.H.S.); elsa.chan@unimelb.edu.au (E.C.C.); 2 Department of Medicine, University of Melbourne, Parkville 3010, Australia

**Keywords:** NOX, oxidative stress, glaucoma, retinal ganglion cells, reactive oxygen species, neurodegeneration

## Abstract

Glaucoma is characterised by loss of retinal ganglion cells, and their axons and many pathophysiological processes are postulated to be involved. It is increasingly understood that not one pathway underlies glaucoma aetiology, but rather they occur as a continuum that ultimately results in the apoptosis of retinal ganglion cells. Oxidative stress is recognised as an important mechanism of cell death in many neurodegenerative diseases, including glaucoma. NADPH oxidase (NOX) are enzymes that are widely expressed in vascular and non-vascular cells, and they are unique in that they primarily produce reactive oxygen species (ROS). There is mounting evidence that NOX are an important source of ROS and oxidative stress in glaucoma and other retinal diseases. This review aims to provide a perspective on the complex role of oxidative stress in glaucoma, in particular how NOX expression may influence glaucoma pathogenesis as illustrated by different experimental models of glaucoma and highlights potential therapeutic targets that may offer a novel treatment option to glaucoma patients.

## 1. Introduction

Glaucoma is a heterogeneous group of disorders unified by loss of retinal ganglion cells (RGCs) and their axons, resulting in a characteristic phenotype consisting of an excavated optic nerve head with corresponding visual field defects. The pathogenesis of glaucoma is incompletely understood with multiple pathophysiological factors and pathways postulated to be causative, such as mechanical, vascular, and immunological factors, leading to the final result of apoptotic death of RGCs [1]. Increasingly it is becoming apparent that these factors do not contribute to glaucoma independently, but rather they can be viewed as a continuum in which each factor contributes to the damage of retinal ganglion cell axons.

In recent years, the role of oxidative stress has been recognised as playing an important role in the pathogenesis of glaucoma. Oxidative stress is implicated in elevated intraocular pressure [2,3,4] and advancing age [5,6,7] and may explain the underlying process of these risk factors in glaucoma development. In addition to the known correlation of mitochondrial dysfunction to oxidative stress in glaucoma pathology [8], there is accumulating experimental evidence that NADPH oxidase (NOX) can also contribute to oxidative stress in glaucoma. In this article, the role of oxidative stress, in particular the role of NADPH oxidase in the pathogenesis of glaucoma, and potential therapeutic targets related to oxidative stress in glaucoma are reviewed.

## 2. Pathogenesis of Glaucoma

### 2.1. Anatomical Structure of the Optic Nerve Head and Retina

The retina is composed of ten layers that consist of the retinal pigment epithelium as the outermost layer and the internal limiting membrane as the innermost layer. RGCs and their 1.2 to 2.0 million axons reside in the inner retina just external to the internal limiting membrane. All visual information transmitted from the retina to the brain travels through the RGC axons. These unmyelinated axons converge at the optic disc, where they exit the eye after making a 45–90° turn. The optic disc is a circular aperture in the sclera in which a multi-layered network of collagen fibres that insert into the scleral canal wall, known as the lamina cribrosa, allows passage of the axons through its pores. A complex arrangement of supportive glial tissue that consists of astrocytes, other glial cells, capillaries and extracellular matrix coats the meshwork of laminar beams that make up the lamina cribrosa. Past the lamina cribrosa, RGC axons become myelinated and terminate at the lateral geniculate nucleus and superior colliculus in the brain. These sites produce neurotrophins which are then transported in a retrograde fashion to the RGCs. As such RGC axons are critical for maintaining the health of RGCs by regulating the activity and survival of cell somas. 

The blood supply to the optic nerve is complex and unique. The central retinal artery, which enters the eye through the centre of the optic nerve, supplies blood to the inner layers of the retina and sends small branches to only the very superficial layers of the optic nerve head (ONH) [9]. The prelaminar portion of the ONH is supplied by short posterior ciliary arteries that stem off the ophthalmic artery [10,11]. These arteries form an incomplete anastomosis known as the circle of Zinn–Haler, within the scleral canal. From this circle, smaller end-arteries arise to supply the ONH, including branching capillaries that coat the lamina cribrosa. The movement of oxygen and nutrients from the laminar capillaries, through the laminar beam extracellular matrix across the astrocyte basement membrane into the astrocyte, finally reaching the peripheral and central axons of each bundle via cell processes, is critical for maintaining axonal health [12,13].

It is likely that the ONH is the only part of the central nervous system with no blood-brain-barrier as the capillaries lack blood-brain barrier mechanisms [14]. In contrast to retinal circulation, which is highly autoregulated, the ONH circulation is less efficiently autoregulated [15], and susceptible to diffusion from the choroid circulation nearby. It is, therefore, sensitive to circulating molecules such as endothelin-1 and angiotensin II [16], which are known to cause NOX activation [17,18]. 

### 2.2. Mechanical Theory of Glaucoma

The mechanical theory of glaucoma offers a framework to explain the relationship between intraocular pressure-related stress and RGC pathophysiology. Elevated intraocular pressure is a well-recognised risk factor of glaucoma and the only modifiable one. Elevated intraocular pressure is the result of increased resistance in the trabecular meshwork outflow pathway, preventing adequate drainage of aqueous humour. This, in turn, impedes axoplasmic transport, resulting in apoptosis of RGCs [19]. The site of this injury is postulated to occur at the ONH, where RGC axons are more vulnerable to pressure-related changes as lamina cribrosa, being structurally weaker than sclera, is more prone to distortion and posterior-displacement [20,21]. It is the effect of this translaminar pressure gradient on axonal physiology that underpins the mechanical theory of glaucoma. Assessment of the movement of the cribrosal plates in glaucoma have demonstrated that the greatest compressive force is exerted on axons lying in the peripheral part of the optic nerve [21]; this correlates with the clinical observation that vision loss in glaucoma initiates with loss in the peripheral visual field.

However, it is unlikely that gross deformation of the cribrosal plates occurs in the early stages of glaucoma. Whilst axons are undoubtedly damaged and lost in the early stages of glaucoma, it is not due to physical alteration of the lamina cribrosa, and its proposed mechanisms are discussed later in this article. However, stress and strain can build up at the lamina cribrosa due to elevated intraocular pressure [21,22]. Reduced lamina cribrosa elasticity with age means that in older eyes, the lamina cribrosa is less able to return to normal configuration once pressure is normalised [23]. Hence the increased prevalence of glaucoma with increasing age, and ageing is associated with oxidative stress.

### 2.3. Vascular Theory of Glaucoma

The vascular theory of glaucoma postulates that glaucoma is a consequence of insufficient blood supply to the optic nerve due to either elevated intraocular pressure or other risk factors leading to reduced ocular blood flow [24]. This results in hypoxia and ischemia to the RGC and its axons. While the question has been raised of whether reduced ocular blood flow may, in fact, be a *consequence* of elevated intraocular pressure in glaucoma, rather a cause [25], investigators argue that evidence direct us to reduced ocular blood flow as a cause for these reasons: Firstly, effects of reduced ocular blood flow is often more pronounced in those with normal tension glaucoma rather than high-tension glaucoma, such as optic disc haemorrhages [26,27]. Secondly, reduced ocular blood flow is often not confined to the eye alone but often seen beyond the eye [28]. Finally, reduced blood flow precedes glaucomatous disease in at least some patients [29]. There are also many clinical indications of this being the underlying pathophysiology, both within the eye and systemically. Optic disc haemorrhages are one of the hallmarks of glaucoma, especially poorly-controlled glaucoma [26,30]. Increased risk of venous thrombosis [31,32]; and vasoconstriction of the retina [33] are also more frequently seen in patients with glaucoma. Systemically, there is an association between cardiovascular disease and the development of glaucoma. Specific risk factors include systemic hypertension [34,35] or hypotension [36,37,38], a previous haemodynamic crisis [39,40], diabetes [41,42], and increased blood viscosity [43]. Migraines [44,45], Raynaud’s phenomenon [46,47], and other conditions related to vasospasm have also been identified as risk factors for the development of normal tension glaucoma [44,47,48]. In this review, we will focus on the role of NOX-dependent oxidative stress in the eye. However, it is well known that NOX-dependent oxidative stress is implicated in cardiovascular diseases [48]. Impaired endothelial cell function has been described in glaucoma patients [49], as well as increased blood plasma and aqueous humour levels of endothelin-1 [50,51,52], although this finding is not specific to glaucoma patients alone [53].

The exact underlying cause of impaired ocular blood flow still unknown, and a detailed analysis is beyond the scope of this article. However, three potential causes have been suggested: (1) increased resistance to flow such as with arteriosclerosis; (2) reduced perfusion pressure, such as seen in systemic hypotension or primary vasospastic syndrome; (3) increased blood viscosity. The dependence of the ONH on end-arteries to deliver its vascular supply likely predisposes this risk. While we do not yet have a treatment that directly targets reduced ocular blood flow to treat glaucoma, the lowering of intraocular pressure, which is the only currently available treatment strategy, may in part work by increasing blood flow [25]. 

### 2.4. Immunological Theory of Glaucoma 

As with all cell types, homeostasis and survival of RGCs depend on a well-functioning immune system. As glaucoma is frequently considered a neurodegenerative disease, it is perhaps not surprising that processes associated with impairment of the immune system seen in other central nervous system (CNS) diseases are also identified in glaucoma [54]. The idea that glaucoma is associated with impaired immunity was first raised by Wax in 1998 when he described antibodies against endogenous antigens such as heat shock proteins (HSP) in the serum of glaucoma patients [55]. HSPs are important for cellular defence and are upregulated during pathophysiological conditions. Since then, activity associated with both innate and adaptive immunity has been described in glaucoma [56,57,58].

Microglial cells are specialised macrophages of the CNS. They are in the frontline of active immune defence and act as scavengers to clear the debris of dead or dying neurons. However, they can also have a destructive role and can harm the cells by releasing cytokines such as tumour necrosis factor (TNFα) and may play a role in the initiation of RGC death [59]. Microglia have been shown to be activated by elevated intraocular pressure in experimental models of glaucoma [60]. 

Monocytes have been reported to mediate axonal damage in mouse models of glaucoma and inhibition of this activity have been demonstrated to have a protective effect [61]. This is likely to be associated with activation of a leukocyte transendothelial migration pathway which allows proinflammatory cytokines to enter the ONH. Howell et al. [62] subsequently demonstrated that localised radiation of the ONH could be neuroprotective by reducing the activation of optic nerve microglia.

## 3. Oxidative Stress in Glaucoma

Oxidative stress in its simplest form refers to the imbalance of free radicals and antioxidants in the body that can result in tissue damage. Reactive oxygen species (ROS) are a major source of oxidative stress, and they include free radicals such as superoxide anion (O_2_^−^), hydroxyl radical, lipid radical and nitric oxide (NO). Uncontrolled production of ROS can lead to cell damage, necrosis or apoptosis via oxidation of macromolecules such as proteins [63,64], lipids [65], nuclear DNA [66], or mitochondrial DNA [67]. Oxidative stress is recognised to be an important mechanism of cell death in neurodegenerative diseases, including glaucoma [68]. While there are numerous enzyme systems that produce ROS in mammalian cells, four enzyme systems predominate; these include NADPH oxidase [69], xanthine oxidase [70], uncoupled NO synthase [71], and the mitochondrial electron transport chain [72].

Our understanding of the role of oxidative stress generated by the mitochondrial electron transport chain is more profound than the other enzymatic systems because in recent years considerable research has been conducted on the bioenergetic processes of RGC axons [73,74,75], whereas little is known about oxidative stress related to NO synthase and xanthine oxidase in glaucoma. The oxygen consumption of RGCs is immensely high: each RGC consumes 4.68 × 10^8^ ATP molecules/s to prevent complete loss of vision [74]. This is about 5-fold greater than the requirement of mammalian photoreceptors in darkness and similar to the requirement of unmyelinated hippocampal axons (mossy fibres) to maintain action potentials. This high energy expenditure is largely consumed by the generating of action potentials and re-establishing the resting membrane potential [76]. RGCs are able to maintain this considerable energy demand due to the abundance of mitochondria present throughout the cell soma, axon and dendrites. Mitochondria concentration is highest at the lamina cribrosa, and this is consistent with their role in protecting RGCs from damage by ensuring adequate ATP supply, as the lamina cribrosa is a site vulnerable to damage [77]. Mitochondria also play an important role in maintaining synaptic integrity [78].

## 4. NADPH Oxidase and Oxidative Stress

**Figure 1:**
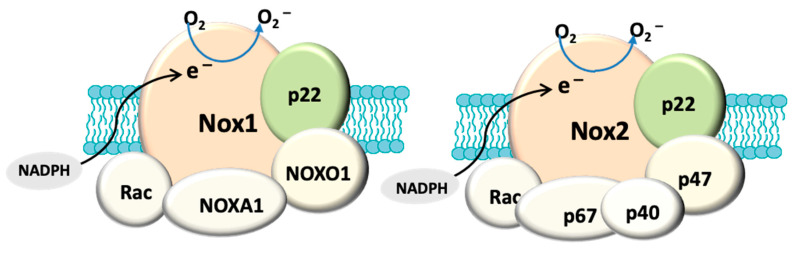
Structural composition of fully assembled NADPH oxidase (NOX). Both NOX1 and NOX2 have a membrane anchoring subunit p22PHOX. NOX1 is associated with cytosolic subunits: NOXA1, NOXO1, Rac and NOX2 is assembled with p67PHOX, p47PHOX, p40PHOX and Rac. The catalytic domain (NOX) allows the transport of electrons (e^−^) from cytosolic NADPH to generate O^2^^−^. In contrast, NOX4 (not shown here) only has p22PHOX. NOX4 is constitutively active and primarily produces hydrogen peroxide.

In addition to mitochondria, NADPH oxidase, also known as the masters of the ROS producing enzymes [79], may also play an important role in the generation of ROS and oxidative stress in glaucoma. There is also evidence that mitochondrial dysfunction can activate NADPH oxidase. For instance, superoxide derived from mitochondria has been shown to induce activation of NOX2 in human lymphoblasts [80]. NADPH oxidase has been identified as one of the major sources of oxidative stress in retinal eye diseases such as ischaemic retinopathy and aged-related macular degeneration [81]. NOX-derived reactive oxygen species (ROS) regulate many cellular processes including proliferation, migration and differentiation [82]. There are seven NADPH oxidase (catalytic subunit NOX [69]) isoforms: NOX1-5 and DUOX1-2. The NOX1, NOX2, NOX4 and NOX5 (NOX5 is only expressed in humans) are widely expressed in vascular and non-vascular cells, and they primarily produce ROS, making them unique from other redox enzymes. These isoforms have been extensively explored in cardiovascular disease, inflammation and fibrosis [81,83] and the role of NADPH oxidase in ocular diseases is still under extensive investigation. Among these isoforms, NOX1, NOX2, NOX4 are the most studied in eye pathology. NOX1 and NOX2 differ from NOX4 in the mode of activation and type of ROS produced [84]. All three isoforms have a membrane anchoring subunit p22PHOX, but the activation of both NOX1 and NOX2 requires association with their cytosolic subunits and NOX4 is constitutively active [85]. NOX1 and NOX2 generate superoxide and NOX4 mainly produce hydrogen superoxide [85] because the latter has an extracytosolic loop that facilitates the spontaneous dismutation of superoxide into hydrogen peroxide [86] (Figure 1). It has been known that the enzymatic activity of NOX can be induced by stimuli. For example, hypoxia has been shown to induce NOX1 expression and ROS production under low glucose condition to cause apoptosis in retinal ganglion cells (RGC) [87]. Proinflammatory cytokine TNFα has also been shown to cause NOX2-dependent ROS production in microglia [88] and TNFα has indeed been detected in aqueous humour of glaucoma patients [89]. HSP that is induced in glaucomatous eye [90] has also been found to regulate the enzymatic activity of NOX1 and NOX2 [91]. Accordingly, all of these stimuli are relevant to glaucoma pathology.

Recently advanced glycation end products (AGEs) that are formed as a result of glycation of proteins and lipids have also been implicated in the pathology of age-related eye diseases such as glaucoma [92,93]. Indeed, restoring the function of AGEs detoxifying enzyme glyoxalase (GLO) has been shown to prevent diabetic retinopathy injury [94]. While AGEs are sources of oxidative stress, the generated oxidant products have been known to promote the formation of AGEs, thus providing a positive feedback mechanism to enhance oxidative stress [92]. Apart from AGEs, NADPH oxidase-derived ROS have also shown to accelerate the synthesis of AGEs. Although it is not the focus of the present review, a correlation in the context of oxidative stress has been proposed among AGEs, NADPH oxidase and mitochondria [95].

## 5. Expression of NADPH Oxidase and Glaucoma Pathology

Several animal models of glaucoma have been used to characterise the expression of NADPH oxidase in the pathogenesis of glaucoma. The animal models utilise different techniques to mimic various pathological processes in glaucoma, such as retinal ischemia and reperfusion, ocular hypertension, and optic nerve crush injury, to mimic pathological features of glaucoma [96]. Some features, including microglial activation and neuronal injury in the retina, and neuronal cell death due to apoptosis of RGC in glaucoma, can lead to vision impairment [97]. Here we describe the relationship between NOX expression and pathological features in the animal models of glaucoma.

### 5.1. Retinal Ischemia and Reperfusion

By applying a transient period of ischemia to the retina to induce neuronal cell injury in mice, Yokota et al. characterised the relationship of NOX expression in neuronal cell death and glial cell activation in ischaemic/reperfused retinas [98]. While the mRNA of NOX1, NOX2, NOX3, NOX4 and NOX cytosolic subunits p22PHOX and p47PHOX is detected in the non-ischaemic mouse retinas, only gene expression of both NOX2 and p22PHOX is induced after ischemia and reperfusion [98]. Furthermore, Yokota et al. [98] used NOX2-deficient mice to verify that NOX2-dependent ROS generation is localised to the inner retina, and NOX2 deletion can prevent both the apoptosis of neurons in the RGC layer and activation of glia after ischemia and reperfusion. In contrast, Dvoriantchikova et al. [87] showed that ischemia and reperfusion enhanced the immunostaining of both NOX1 and its regulatory subunit NOXO1 in the RGC layer without affecting NOX2, NOX4 and p47PHOX in mice. The variations in the time of ischemia and reperfusion or detection methods may account for the discrepancies between the two studies. Although both studies subjected the retinas to forty to sixty minutes of ischemia, the reperfusion duration was different [87,98]. It should be noted that Yokota et al. [98] used Western blotting to analyse the overall expression of NOX2 in the retinas and showed that NOX2 induction commences at three hours and peaks at six and twelve hours after ischemia and reperfusion [98]. On the other hand, Dvoriantchikova et al. used immunofluorescence to determine the tissue distribution of NOX expression in retinal sections from mice following three hours of retinal ischemia and reperfusion [87]. The two study outcomes also reflect the distinct activation of NOX isoforms in different layers of the retina upon ischemic challenge, for example, NOX1 is mainly induced in the RGC layer where it contributes to the death of RGC [87] and NOX2 is distributed throughout the inner retina [98] where it promotes glial cell activation as well as RGC apoptosis. Hence inhibiting NOX activation may prevent neuronal cell damages resulting from the ischaemic insult that occurs in acute glaucoma. 

### 5.2. Ocular Hypertension

Ocular hypertension in animals is induced by either applying laser photocoagulation to the trabecular meshwork or cauterizing the episcleral vein [96]. NOX2 has been found to co-localise with activated microglia in the ONHs from mice after laser photocoagulation of the trabecular meshwork [73]. It is proposed that the ROS produced from the microglial NOX2 cause a disruption to the axonal transport in these mice following laser photocoagulation to the trabecular meshwork [73]. In a model of unilateral ocular hypertension induced by episcleral vein cauterization, ocular hypertension causes activation of both astrocytes and microglia in retinas, and this is accompanied by an induction of NOX2 mRNA [99], supporting a role of NOX2 in retinal inflammation. Although the expression of NOX2 is assessed in the two models of ocular hypertension, the interaction between NOX2 and microglial activation has not been investigated [73,99]. Interestingly, NOX2 in microglia is thought to promote the polarization of microglia to the M1-like phenotype in mice brain after traumatic brain injury because the deletion of NOX2 gene reduces M1-like activation but induces the activation of M2-like phenotype in the injured brain [100]. M1-like phenotype is known to be involved in inflammatory response while M2-like microglia facilitates tissue repair by suppressing injury-induced inflammation and re-establishing tissue homeostasis [101]. The demonstration of NOX2 in polarizing microglia to M1-phenotype after brain injury reflects a major role of NOX2 in neuroinflammation. Moreover, NOX2-dependent ROS signalling can promote leukocyte transendothelial migration [102], a process that is known to allow proinflammatory cytokines to enter the ONH during inflammation. Apart from the degeneration of RGC, neuroinflammation also contributes to the progression of glaucoma [89]. Hence, these data provide some insights into how NOX2 may facilitate the initiation and propagation of inflammation. Because microglial activation can induce or amplify the damages to RGC in glaucoma [103], it will be of great interest to elucidate whether NOX2 also affects the polarisation of microglia or leukocyte transendothelial migration in animal models of glaucoma. Apart from its association with activated microglia, NOX2 expression has also been found in retinal arterioles of mice with ocular hypertension [104]. The mRNA level of NOX2 but not NOX1 is upregulated in retinas with ocular hypertension, and it is thought that NOX2-dependent ROS production is involved in the reduced endothelium-dependent relaxation of retinal blood vessels in the presence of ocular hypertension [104]. In a comparable microvascular network to the eye such as the cerebrum, NOX2 is also involved in the angiotensin II-induced endothelial dysfunction [105], and angiotensin II is known to cause hypertension [106]. The findings thus highlight the role of NOX2 in endothelial dysfunction induced by hypertension.

### 5.3. Optic Nerve Injury

Optic nerve crush is another experimental approach that induces neuronal cell damage in the retina [107]. Mice with optic nerve crush injury demonstrated RGC death as well as increases in mRNA expression of NOX1, NOX2 and NOX4 in the retina [108]. *In situ* detection of ROS also confirmed that the retinal ganglion layer is the primary site of ROS production in these mice [108]. Since ROS are short-lived, RGC layer presumably harbours the identified ROS generating enzymes NOX1, NOX2 and NOX4, which promote RGC death through ROS production after optic nerve crush injury [108]. In a rat model of nonarteritic anterior ischemic optic neuropathy where the optic nerve was injured with laser, it was thought that NOX2 induction is involved in the microglial activation in the anterior optic nerve [109]. 

Accumulating findings from animal models of glaucoma revealed that NOX induction is involved in the progression of glaucoma (Table 1); however, the expression profile of NOX in human eyes is very scarce, and this is largely due to very limited tissue sources. The demonstration of NOX2 and NOX4 induction in alkali-burnt eye sections from a patient is one of the few investigations that characterises NOX expression in human corneal specimen [110]. Another assessment of NOX in clinical samples comes from the profiling of NOX4 protein expression in different grades of excised ocular tumours and, NOX4 expression is found to be correlated to higher grade retinoblastoma and massive choroidal invasion [111]. The profiling of NOX expression in clinical samples from glaucoma patients is yet to be explored. 

## 6. Targeting Oxidative Stress for Glaucoma

### 6.1. Antioxidant Therapy

Several clinical studies have used different outcome measures to assess the protective effects of antioxidants in glaucoma patients. Falsini et al. [112] evaluated the effect of antioxidant epigallocatechin-gallate (EGCG) on the retinal function of ten patients with open-angle glaucoma using pattern electroretinogram (PERG), and the study showed that oral EGCG supplement given over three months slightly improved the patients’ inner retinal function. Since PERG measurements correlate with the activity of RGC, it is thought that short-term EGCG treatment delays the regression of glaucoma-induced damages on RGC to improve inner retinal function [112]. It has also been known that glaucomatous eyes have impaired ocular blood flow [113] that would progressively lead to an ischaemic environment which can compromise the physiology of the retina. Recently, open-angle glaucoma patients receiving a month of mixed antioxidants, including vitamins C, E, B6 and B12 and herb remedy like Gingko Biloba, show better ocular blood flow parameters, such as increases in supero- and infero-temporal retinal capillary mean blood flow and decreases in retinal vessel resistance when compared to placebo [113]. In contrast, a separate cohort of fifty-four patients taking oral antioxidant supplement, mainly consisting of vitamins A, B, C, E, lutein and essential minerals such as zinc and selenium, showed no differences in visual acuity and thickness of both peripapillary retinal nerve fibre and macular ganglion cell complex when compared to placebo [114]. Because the three studies used different antioxidant formulations and performed different outcome measures, it is difficult to rationalise the discrepancies. Nevertheless, there is still a lack of large clinical trial to draw any solid conclusion on the neuroprotective effect of antioxidant therapy in glaucoma. As such, a study has recently commenced and planned to recruit 612 patients to compare the neuroprotective effect of an ophthalmic solution of antioxidants Coenzyme q10 and vitamin E to placebo with a follow-up of three years [115]. The study outcome will clarify if antioxidants can slow down glaucoma progression based on the examination of a patient’s visual field.

### 6.2. Pharmacological Inhibition of NADPH Oxidase

While antioxidants are being trialled in glaucoma patients, preclinical research in animals has also been performed to assess if targeting the source of ROS production is an alternative approach for controlling oxidative stress. This concept comes as some earlier antioxidant trials have produced dissatisfactory outcomes when assessing the progression of vascular diseases [116]. Based on a literature review on the clinical trials of vitamins between 1981 and 2005, it is thought that some antioxidant vitamins like vitamin E, when administered alone, produces adverse effects by acting as a prooxidant and soaking up the endogenous pool of antioxidants in the already vulnerable oxidative stress environment [116,117]. Consequently, later trials tend to use a combination of antioxidant vitamins to avoid the harmful effects of vitamin monotherapy. Furthermore, high doses of antioxidants are often required to neutralise such a high level of ROS in diseased conditions [116]. As such, targeting the source appears to be more effective. 

Pharmacological inhibition is one of the approaches that is commonly used to block the contribution of NADPH oxidase and has been widely explored in an animal model of vascular diseases [118] and more recently in retinal pathological conditions such as ischaemic retinopathy and retinal inflammation [119,120]. There are however very limited studies on the pharmacological intervention of NOX in animal models of glaucoma and the beneficial effects of NOX inhibition have only been demonstrated with a non-selective NADPH oxidase inhibitor apocynin and NOX2 deficient mice [98]. As such, it is important to assess the protective effect of NOX inhibitor in an animal model of glaucoma. A panel of NOX inhibitors such as GKT137831, GSK2795039 and GLX7013114 have been developed to selectively target the various isoforms of NOX as different NOX subtypes tend to be involved in various pathological conditions in the vascular systems [121], for example, NOX2 in inflammation and NOX4 in fibrosis. In eye pathologies, a dual inhibitor of NOX1 and NOX4, GKT137831 has been assessed for its protective effects in animal models of ischaemic retinopathy and retinal inflammation and retinal endothelial cells under conditions simulating diabetes [120,121,122,123]. GKT137831 is developed by GenKyoTex and has been assessed in a variety of animal models, including kidney diseases and liver fibrosis [118,124] prior to the evaluation in eye diseases. GKT137831 (also known as setanaxib) is currently in a clinical trial for diabetes and kidney diseases [124]. Of all the developed NOX inhibitors, setanaxib is the first of its kind to be recognised and categorised into the newly World Health Organisation (WHO) approved stem naxib, or NADPH oxidase inhibitors [124]. 

Like setanaxib, GSK2795039 is a small molecule developed for NOX2 [122] and appears to stand for its claim when it is recently challenged for its selectivity using in vitro NOX evaluation assays [121]. The potency of GSK2795039 has been shown in animal models of inflammation such as Freund’s adjuvant-induced inflammation in the paw and acute pancreatitis [122], and cell cultures of inflammatory cells like neutrophils [125] and monocytes [126]. Recently, GSK2795039 has also demonstrated benefits in neuronal injury [127]. A single dose of GSK2795039 given prior to induction of traumatic brain injury in mice improves neurological deficit scores and prevents the breakdown of the blood brain area at twenty-four hours after injury [127]. GSK2795039 treatment has been found to suppress NOX2-dependent ROS generation in the neurons cultured from the GSK2795039-treated injured cerebral cortex, thereby preventing cell survival and promoting cell growth [127], suggesting that the beneficial effects relate to NOX2 inhibition. Glucox Biotech has developed a series of highly specific NOX4 inhibitors such as GLX351322 [128] and GLX7013114 [129], and the latter has shown anti-NOX4 activity in preventing the transition of lens cells into a phenotype that actively produces scar proteins following stimulation with TGFβ1 [129]. Interestingly, a single intravitreal injection of GLX7013114 suppressed the accumulation of microglia and glial cell activation in the retinas from rats with AMPA-induced retinal excitotoxicity [130], highlighting the neuroprotective effect of GLX7013114. 

## 7. Future Horizons

**Figure 2:**
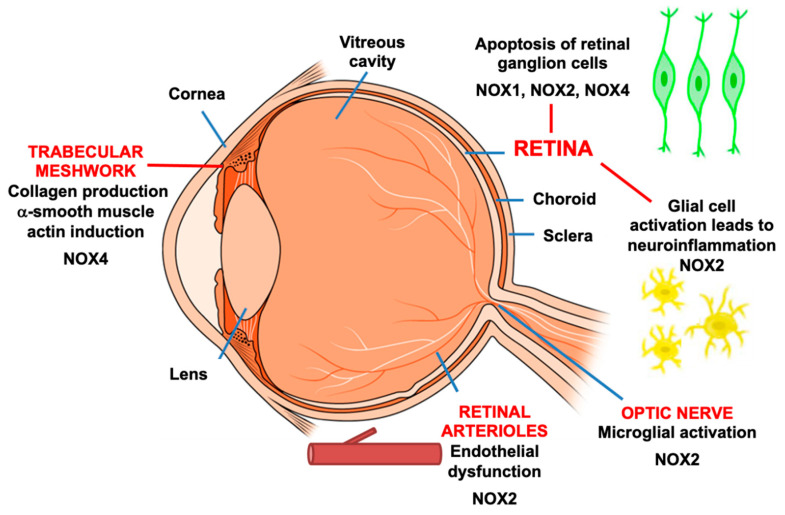
Expression of NADPH oxidase in various tissue compartments of the eye. Experimental models of glaucoma provide evidence that NOX induction contributes to cellular processes that are important in the progression of glaucoma.

In addition to having direct effects on RGCs and the retina in glaucoma, there is early evidence that NADPH oxidase may be implicated in the pathophysiology of glaucoma through other anatomical pathways (Figure 2). Alterations in extracellular matrix remodelling at the trabecular meshwork resulting in elevated intraocular pressure can contribute to glaucoma development [131]. Transforming growth factor (TGFβ) has been characterised as an important contributing factor to glaucoma pathophysiology. For example, TGFβ has been demonstrated to cause cell apoptosis in cell cultures and promote collagen synthesis in the trabecular meshwork; it also elevated intraocular pressure in a perfused organ culture model using human donor anterior segments [131,132]. Importantly, elevated concentrations of TGFβ have been detected in aqueous humour from patients with glaucoma [131], implicating its role in glaucoma. Interestingly NADPH oxidase has been identified as a potential source of ROS generation at the trabecular meshwork under chronic stress [133]. Some preliminary works have been recently performed in human trabecular meshwork cells to assess the relationship between TGFβ fibrotic responses and NOX expression [134,135]. TGFβ1 is found to stimulate the gene expression of NOX4 and fibrosis markers such as collagen and α-smooth muscle actin [134] while TGFβ2 selectively induces NOX4 expression without affecting NOX1–NOX3 and NOX5 [135]. Our group has also previously shown that TGFβ1 activates NOX4 to promote collagen synthesis in Tenon’s fibroblasts [136], which are major contributors to fibrosis in pathological wound healing [137]. These findings support NOX4’s involvement in fibrotic responses, but further research is required to dissect out the role of NOX4 and other NOX isoforms in regulating extracellular matrix turnover in the trabecular meshwork. 

Vascular dysregulation in the eye is another pathological feature of glaucoma, and the underlying mechanism is still unclear. This phenomenon may be partly attributable to the impaired autoregulation and endothelial dysfunction under the influence of high intraocular pressure and appears to be involved in oxidative stress [79]. While further investigation is required to delineate the relationship between oxidative stress and impaired endothelial function in ocular hypertension, a vascular reactivity study conducted in hypertensive patients may shed some lights [138]. Accordingly, the impaired endothelium-dependent relaxation of the coronary artery in hypertensive patients is due to a reduction of bioavailable vasodilator NO [138]. It should be noted that when ROS such as superoxide (O_2_^−^) is produced in close proximity to NO, the two labile ROS can react to produce the more damaging peroxynitrite (ONOO^−^) [139] if endogenous antioxidants like superoxide dismutase are compromised—as such, controlling oxidative stress in glaucoma may restore the local pool of NO and preserve endothelial function. Therefore, the implication of NOX-dependent oxidative stress in hypertension-induced endothelial dysfunction should be investigated further in an in vivo setting. The feasibility of delivering vessel constricting/dilating agent to the retina would present a challenge even though a variety of techniques such as laser Doppler flowmetry and optical coherence tomography are available for assessing live retinal activity including blood flow in the eye [140]. 

The profiling of NOX expression in samples from patients with glaucoma presents a challenge to the research field due to tissue availability. This problem may be overcome with the advent of next generation sequencing technology such as RNA-seq. The main advantage of RNA-seq is its capability of producing a variety of information such as gene isoforms, alternative splice sites and allele-specific expression from a small sample of cDNA [141]. Indeed RNA-seq has already been applied in ophthalmology research [142] and RNA-seq has discovered important genes that are crucial to the development of eye diseases including retinitis pigmentosa [143,144], glaucoma and keratoconus [145]. Indeed Donato et al. [146] recently used RNA-seq and discovered that damaged mitochondrial DNA plays a significant role in retinal degeneration.

## 8. Conclusions

While oxidative stress is increasingly recognised to play an important role in the pathogenesis of glaucoma, the research spotlight has been on mitochondrial dysfunction as the main source of this oxidative stress. This review has provided experimental evidence that NADPH oxidase is an emerging source of oxidative stress in glaucoma, and its induction plays a role in the progression of glaucoma. Targeting NADPH oxidase appears to be a viable strategy to control neuroinflammation and delay or prevent the degeneration of RGCs, but further research is required to understand its interaction with other enzymatic sources, such as the mitochondria. We have discussed the expression NADPH oxidase in different models of glaucoma, highlighted the potential role of antioxidant therapy targeting NADPH oxidase and other enzymatic pathways in glaucoma, and identified prospective areas of research interest implicating NADPH oxidase in glaucoma.

Despite the recent advances made in glaucoma treatment, the only proven therapeutic target is still through the lowering of intraocular pressure to slow the loss of RGCs and their axons. Antioxidant therapy targeting NADPH oxidase presents a favourable alternative treatment for glaucoma patients through the neuroprotection of retinal ganglion cells and their axons, in addition to pressure lowering medication. Whilst the majority of this research is still in preclinical phases, it offers an exciting new field of research in glaucoma.

## Figures and Tables

**Table 1:** Summary of NOX expression in animal models of glaucoma.

Animal Model	Tissue Basal Expression of NOX	Tissue NOX Induction upon Injury	Localisation of NOX	Site of ROS Production	NOX Association to Retinal Injury
Retinal ischemia and reperfusion (6 h) [98]	mRNA: NOX1, NOX2, NOX3, NOX4, p22PHOX and p47PHOX in retinas	mRNA: NOX2 and p22PHOX in retinas	Not performed	Inner retina	Apoptosis of retinal ganglion cells Glial cell activation
Retinal ischemia and reperfusion (3 h) [87]	Protein: NOX1, NOX2, NOX4, NOXO1 and p47PHOX in retinas	Protein: NOX1 and NOXO1 in retinas	Retinal ganglion cells	Inner retina	No evaluation of cell injury in the eye section
Optic nerve crush [108]	mRNA: NOX1, NOX2, NOX4 in retinas	mRNA: NOX1, NOX2, NOX4 in retinas	Not performed	Retinal ganglion layer	Reduction in the survival of retinal ganglion cells
Photocoagulation of trabecular meshwork [73]	NOX2 mRNA and protein in optic nerve head	NOX2 mRNA and protein in optic nerve head	Microglia in optic nerve head	Optic nerve head	Microglial activation in optic nerve head
Cauterization of episcleral vein [99]	NOX2 mRNA in retinas	NOX2 mRNA in retinas	Not performed	Not measured	Microglial activation in retinas
Cauterization of episcleral vein [104]	NOX1 mRNA NOX2 mRNA and protein in retinas	NOX2 mRNA and protein in retinas	Retinal ganglion layer Retinal arterioles	Retinal ganglion layer Retinal arterioles	Impaired endothelial function
Laser-induced injury on optic nerve [109]	NOX2 protein in optic nerve	NOX2 protein in optic nerve	Not performed	Not measured	Microglial activation in the optic nerve

## Abbreviations

AGEs	Advanced glycation end products
CNS	central nervous system
EGCG	epigallocatechin-gallate
GLO	Glyoxalase
HSP	heat shock protein
NO	nitric oxide
NOX	NADPH oxidase
O_2_^−^	superoxide
ONH	optic nerve head
ONOO^−^	peroxynitrite
PERG	pattern electroretinogram
RGC	retinal ganglion cell
TGFβ	transforming growth factor β
TNFα	tumour necrosis factor α
